# The cerebrospinal fluid immune microenvironment in lung cancer leptomeningeal metastasis: cellular ecosystems and therapeutic possibilities

**DOI:** 10.3389/fimmu.2026.1959655

**Published:** 2026-09-30

**Authors:** Yuanzheng Zhao, Haitao Guo, Yongchuan Guo

**Affiliations:** 1 Department of Neurosurgery, Jilin Cancer Hospital, Changchun, Jilin, China; 2 Department of Neurosurgery, The Second Norman Bethune Hospital of Jilin University, Changchun, Jilin, China

**Keywords:** cerebrospinal fluid, immune microenvironment, leptomeningeal metastasis, lung cancer, tumor-immune crosstalk

## Abstract

Leptomeningeal metastasis (LM) is a devastating form of central nervous system (CNS) dissemination in lung cancer, particularly non-small cell lung cancer (NSCLC). Current treatment frameworks emphasize CNS drug penetration, oncogenic driver evolution, and intrathecal therapy. Emerging evidence supports viewing the cerebrospinal fluid (CSF) compartment as an active tumor-immune space, but the field remains early, with limited lung cancer-specific mechanistic and single-cell datasets. Lung cancer cells in CSF appear to adapt to low protein content, limited nutrients, fluid shear, weak matrix support, and compartmental immune surveillance. Meanwhile, available CSF studies suggest immune states marked by exhausted T cells, regulatory lymphoid programs, suppressive myeloid remodeling, cytokine dysregulation, and tumor-supportive signaling. This review summarizes the CSF metastatic niche, tumor adaptation, immune-cell states, tumor-immune crosstalk, therapy-associated remodeling, and CSF multi-omics in lung cancer LM. We propose that CSF-guided profiling may help generate immune-state classifications and inform future targeted, intrathecal, immune, or combination strategies, while requiring prospective validation before routine treatment guidance.

## 1 Introduction

Leptomeningeal metastasis (LM) is one of the most devastating patterns of central nervous system (CNS) dissemination in lung cancer. It occurs when malignant cells colonize the leptomeninges, subarachnoid space, cerebrospinal fluid (CSF), cranial nerves, spinal roots, or ventricular surfaces, producing a diffuse compartmental disease that is difficult to diagnose, monitor, and treat (1–14). Non-small cell lung cancer (NSCLC), particularly oncogene-driven adenocarcinoma, is a major source of LM because improved systemic control and prolonged survival have increased the number of patients at risk for CNS progression (1). Despite advances in epidermal growth factor receptor tyrosine kinase inhibitors (EGFR-TKIs), anaplastic lymphoma kinase (ALK) inhibitors, immune checkpoint blockade (ICB), radiotherapy, and intrathecal therapy, the prognosis of lung cancer LM remains poor and neurological morbidity is substantial (1–3).

Three classical frameworks have long guided the clinical understanding of NSCLC leptomeningeal metastasis: CNS drug permeability, intratumoral oncogenic evolution, and compartment-specific cytotoxic intervention. All three perspectives retain clinical value, but they may not fully explain heterogeneous patient outcomes. The blood-brain and blood-CSF barriers limit systemic drug delivery to the CSF compartment. Meanwhile, CSF ctDNA can capture resistance mutations that are not detected in peripheral blood, and intrathecal chemotherapy delivers cytotoxic therapy directly to intrathecal tumor cells (2, 3, 15, 16). These explanations, however, do not account for all clinical variation observed in practice. Patients with similar driver mutations and treatment histories may show different neurological responses, CSF progression patterns, and sensitivity to immunotherapy. LM also differs from parenchymal brain metastases (BrMs), even when both arise from lung cancer. A model focused mainly on drug delivery risks reducing CSF to a pharmacologic sanctuary, whereas a tumor-genomics-only model may underrepresent host-cell states. A more balanced perspective should therefore consider CSF as an active disease compartment in which malignant cells interact with CNS border tissues, immune cells, soluble mediators, and treatment-related selective pressure.

Recent single-cell, multi-omic and mechanistic studies support a more nuanced view of lung cancer LM as a CSF-associated tumor-immune process rather than simply an extension of systemic metastasis, although the evidence base remains limited and uneven. Cancer cells that enter CSF must survive in a low-protein, low-cellularity, nutrient-constrained fluid and may reshape the host compartment to support growth (17, 18). Tumor-derived complement component 3 (C3), for example, can disrupt the blood-CSF barrier and allow plasma mitogens to enter CSF, indicating that malignant cells can remodel this niche (18). CSF from LM has been reported to contain malignant cells, exhausted or dysfunctional T cells, regulatory lymphoid states, and suppressive macrophage programs, with immune signatures that may differ from blood and parenchymal CNS lesions (19–22). A central unresolved question is not only whether immune cells can enter CSF, but whether the local compartment allows them to remain functional.

This immune-compartment view has potential therapeutic implications, but these should be treated as hypotheses for study design rather than established treatment rules. In a tumor-dominated, immune-cold CSF environment, rapid reduction of tumor burden with CNS-penetrant targeted therapy or intrathecal chemotherapy may be prioritized before immunotherapeutic intervention. If clonally expanded cytotoxic T cells are present in CSF, ICB or intrathecal immunotherapy may have a stronger biological rationale, although clinical confirmation remains limited. In contrast, suppressive myeloid programs may inhibit T cell activity from the beginning; in such cases, macrophage-tumor feedback, aberrant vascular permeability or cytokine circuits may need to be considered as combination targets (20, 22–24). These states cannot be distinguished reliably by plasma biomarkers alone. CSF circulating tumor DNA (ctDNA), CSF circulating tumor cells (CTCs), single-cell RNA sequencing (scRNA-seq), T cell receptor (TCR) profiling, cytokines, proteomics, and serial sampling may provide a disease-proximal platform for exploratory immune-state classification and adaptive monitoring (15, 16, 23, 25). The practical goal is to define reproducible CSF states that can be tested prospectively for treatment selection and response assessment.

This review examines the CSF immune microenvironment in lung cancer LM, with emphasis on cellular states, evidence boundaries, and therapeutic hypotheses. The organizing view is not that CSF contains a fully mapped ecosystem, but that available data repeatedly point to a limited set of testable axes: malignant-cell adaptation to the CSF, myeloid-dominant immune suppression, exhausted or ineffective lymphoid immunity, soluble barrier-inflammatory signaling, and CSF-based molecular monitoring. We first discuss the metastatic niche, including barriers, CSF flow, meningeal lymphatics, immune surveillance, and spatial heterogeneity. We then examine how lung cancer cells may adapt to CSF through survival, metabolic, adhesive, complement-associated, and immune-evasive programs. The next sections focus on immune states, including T cell dysfunction, regulatory lymphoid states, myeloid remodeling, and inflammatory but non-protective immunity. We then link tumor-immune crosstalk to therapy-associated remodeling, CSF multi-omics, and CSF-guided precision therapy as a future research direction. The central argument is that lung cancer LM should be studied through concrete CSF measurements rather than broad ecological labels: tumor burden by ctDNA or CTCs, malignant and immune-cell states by scRNA-seq, TCR clonality, myeloid programs, and cytokine or protein markers.

## 2 The CSF metastatic niche

### 2.1 Anatomical barriers and routes of dissemination

LM from NSCLC develops in an anatomical compartment that is distinct from both the lung primary tumor and parenchymal BrMs. In clinical practice, LM is defined by malignant involvement of the leptomeninges, subarachnoid space, CSF, cranial nerves, spinal roots, or related ventricular surfaces (1–4). This definition is useful because it places the disease in a fluid-filled border compartment rather than in a conventional solid-tumor bed. The CSF compartment is bounded by the pia, arachnoid, ependyma, choroid plexus, and meningeal vasculature. These structures create interfaces that can restrict, filter, and redirect the movement of cells, proteins, metabolites, and drugs (2, 17). In the case of lung cancer cells, it is just the beginning to reach the CNS. They also need to adjust to the CSF, a compartment that has minimal stromal support, and soluble growth factors are relatively dilute, and immune surveillance is focused at CNS borders as opposed to being dispersed throughout a dense tissue matrix (17, 18).

The pathways for the dissemination of lung cancer cells to the leptomeningeal-CSF compartment are not fully understood. Tumor cells can reach CSF either clinically and pathologically via hematogenous seeding of meningeal vessels, parenchymal BrMs invading into CSF spaces, perivascular or perineural spread, or through the choroid plexus and ventricular surfaces (1–3, 17). These routes may be present in a single patient. This is reflected in the 2023 European Association of Neuro-Oncology and European Society for Medical Oncology (EANO-ESMO) guideline. LM presents with different patterns, MRI appearances and CSF cytology (2). The EANO-ESMO classification separates confirmed cytology-positive disease from probable and possible LM, as well as linear, nodular, mixed and MRI-negative patterns (2, 4). This classification is also relevant for immunology of brain tumors. Such variations may result in vastly different exposures of tumor cells to CSF flow, meningeal blood vessels, local stroma and immune-cell infiltration.

In a mechanism-based review, it is critical to differentiate between anatomical entry and ecological colonization. Accessing a meningeal vessel does not imply that a cancer cell has effectively established LM. It still needs to endure in low-mitogen fluid environment, bind to CSF-exposed surfaces and avoid immune surveillance at CNS borders. This difference can be used to understand why the CNS is frequently affected in late-stage lung cancer, but only a minority of patients have overt LM. It also explains the reason why radiographic results, CSF cytology and CSF molecular tests detect overlapping, yet not identical disease states (2–4, 15, 16). As an illustration, nodular meningeal enhancement can be indicative of a pattern of tumor growth with restricted access to the CSF whereas diffuse linear enhancement with numerous CSF CTCs can indicate more widespread communication with the fluid compartment. The pathway through which tumor cells infiltrate and disseminate within the meninges might thus influence not only what is detectable in clinical specimens, but also the effectiveness of treatment in reaching the disease.

### 2.2 CSF flow, nutrient constraints, and cellular sparsity

One of the characteristics of the CSF metastatic niche is fluid dynamics. The choroid plexus produces most of the CSF, circulates it through the ventricular system and subarachnoid space, and exits the compartment via arachnoid granulations, meningeal lymphatic vessels, and other drainage pathways (26–30). This circulation may transport tumor cells across craniospinal axis and nerve roots to cause multifocal neurological symptoms and lesions that are spatially unrelated. But the same flow also diffuses soluble mediators and subjects malignant cells to shear forces and the strains of long-term suspension. LM thus tends to be a diffuse disease of the whole CSF compartment even in cases where imaging reveals only focal nodules or few dominant symptomatic sites (1–4).

LM is also distinguished by the biochemical composition of CSF, as opposed to parenchymal BrMs. Normal CSF is relatively sparsely cellular and has a low protein or many of the growth factors needed to support tumor expansion compared with plasma and solid-tumor interstitial fluid (17, 18, 29). Boire et al. demonstrated that cancer cells that were selected to grow in leptomeningeal upregulated C3. C3a receptor signaling in the choroid plexus epithelium was then activated by tumor-derived C3, which compromised the blood-CSF barrier and allowed plasma mitogens to enter the CSF (18). Their results changed the perspective of CSF as a passive channel to a metabolically hostile environment that malignant cells have to actively remodel. Effective growth thus requires both tumor cell and host border tissue changes to be coordinated. In this regard, lung cancer LM is not merely an issue of dissemination, but also niche construction.

### 2.3 Meningeal lymphatics and immune surveillance

The old concept of CNS immune privilege has been altered significantly. Organized populations of immune cells and established drainage routes are now recognized to exist in the meninges, dural sinuses, choroid plexus, and CSF spaces (26–30). Macromolecules and immune signals at the borders of the CNS are transported by meningeal lymphatic vessels to the deep cervical lymph nodes, but the choroid plexus is both an epithelial barrier and an immune interface (26–30). This is specifically applicable to LM, since tumor cells lodge in areas where immune surveillance, antigen drainage and leukocyte trafficking converge. The leptomeningeal space is thus not devoid of immune activity. It can be more accurately described as an immune-specialized compartment, containing fewer resident cells and stricter regulations of cellular entry than peripheral tissues.

Special attention should be paid to the choroid plexus. It constitutes the blood-CSF barrier (BCSFB), secretes CSF, reacts to inflammatory signals and contains stromal macrophages, dendritic cells (DCs) and T cells that mediate immune surveillance and cellular trafficking (29, 30). This interface is a gateway as well as a target in LM. The factors secreted by tumor cells may enhance BCSFB permeability, whereas inflammation and treatment can alter the populations of immune-cells that can access the CSF (18, 29, 30). Another active participant in LM is the dura. Recent multi-omic and imaging research discovered that macrophages derived by the dura are able to invade the CSF during LM and induce immunosuppression and invasion of the barrier via a secreted phosphoprotein 1-matrix metalloproteinase 14 (SPP1-MMP14)-related axis (20). These results directly relate meningeal border immunity to tumor progression. They also indicate that the immune landscape of LM starts forming prior to complete colonization of the CSF by tumor cells.

### 2.4 Differences from brain parenchymal metastasis and peripheral blood

LM should not be treated as a liquid extension of parenchymal BrM. This distinction is supported at different evidence levels. Clinical guidelines and consensus reviews establish that LM requires compartment-specific diagnosis, staging, treatment, and response assessment (2, 3, 17). Direct lung cancer CSF studies further show that CSF cell-free DNA and CSF tumor-derived DNA can capture compartmental tumor evolution that may be missed in plasma (15, 16). At the cellular level, however, the lung cancer LM evidence remains more limited and is mainly derived from early CSF scRNA-seq or CSF-CTC studies and recent NSCLC BrM-LM comparisons (19, 21, 22, 31, 58). Parenchymal BrMs grow within brain tissue and interact with astrocytes, microglia, neurons, endothelial cells, pericytes, and extracellular matrix, whereas LM develops in a fluid and meningeal-border compartment where tumor cells, immune cells, CSF proteins, and barrier surfaces are the main elements. Peripheral blood may reflect systemic immunity and tumor burden, but it often fails to capture local CSF tumor evolution, immune-cell states, or cytokine circuits (15, 16).

Recent studies reinforce this compartmental distinction, but they should not be interpreted as a single homogeneous LM evidence set. Clinical and radiographic LM studies support the prognostic and measurement relevance of MRI pattern, CSF cytology, radiographic burden, CSF CTCs, and CSF cfDNA in solid-tumor or lung cancer LM (34, 37). Studies of BrM with CSF-space invasion or NSCLC BrM cohorts help explain how parenchymal lesions may precede or predict overt LM, but they address risk and transition rather than the established LM immune microenvironment (35, 36). Single-cell, spatial, and immune-profiling studies of lung cancer BrM or driver-mutated NSCLC provide useful comparative context for brain-tissue metastatic niches, but they should be separated from direct LM evidence (38–42). Similarly, melanoma LMD spatial transcriptomics provides cross-cancer leptomeningeal context rather than lung cancer-specific LM evidence (43). Direct CSF evidence in lung cancer LM comes primarily from CSF profiling of breast and lung cancer LM, lung adenocarcinoma CSF-CTC single-cell analysis, NSCLC BrM-LM comparison, and LUAD-LM CSF TROP2 profiling (19, 21, 31, 58). Together, these data suggest that the immunological difference between BrM and LM is not simply quantitative, but the conclusion remains strongest when direct lung cancer LM studies are distinguished from BrM or cross-cancer evidence.

### 2.5 Linear versus nodular LM as spatially distinct niches

The difference between linear and nodular LM might be more than a radiographic pattern. Linear LM typically reflects the spread of tumor cells along the CSF-exposed leptomeningeal surfaces, in which malignant cells are directly exposed to the fluid compartment. This renders them easier to detect by cytology and liquid biopsy and, in theory, easier to access by intrathecal therapy (2–4). Nodular LM can act differently. Its more structured local architecture, its closer association with blood vessels and its increased reliance on attachment to the matrix may make it look like a metastasis of the meningeal border tissue, as opposed to a process that occurs entirely in the fluid phase. These patterns frequently coexist, indicating that one patient can have multiple different ecological states simultaneously.

There are also practical consequences of this spatial heterogeneity for immunity. In linear, CSF-predominant disease, tumor and immune cells are exposed to highly diluted cytokines, limited antigen presentation sites and a higher dependence on soluble survival signals in the CSF. Nodular lesions may provide a different environment with more intimate stromal and vascular interactions that affect immune-cell sequestration, checkpoint expression, drug uptake and radiographic appearance. Existing clinical classifications already combine imaging with CSF cytology (2, 4). The challenge now is to understand how these patterns correlate with different CSF immune states, tumor-cell phenotypes and therapeutic vulnerabilities. **Figure 1** illustrates the CSF niche accordingly: not as a homogeneous space, but as a barrier-controlled compartment with fluid flow, immune surveillance and significant spatial variation.

**Figure 1:**
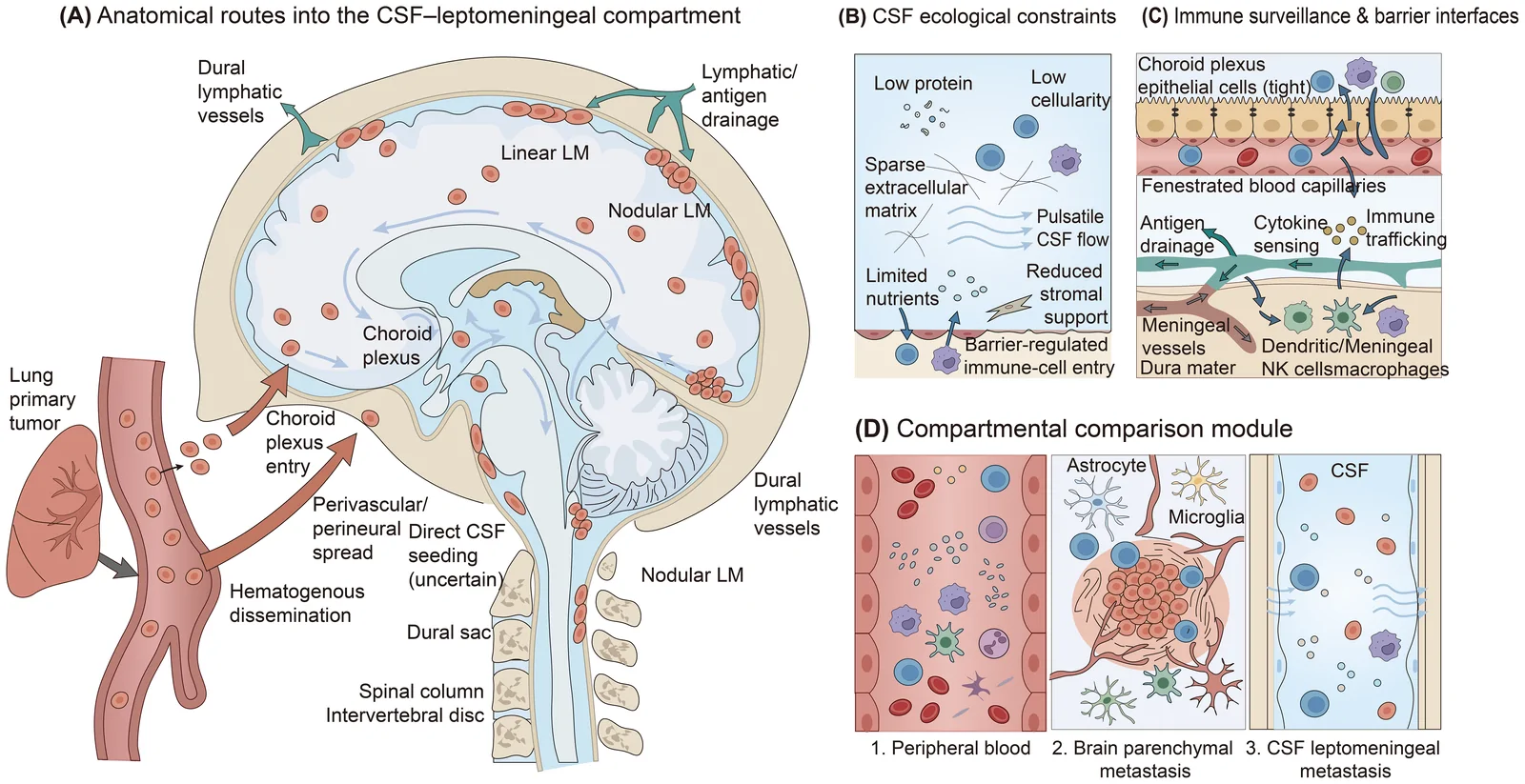
Lung cancer leptomeningeal metastasis emerges within a barrier-regulated cerebrospinal fluid niche. **(A)** Anatomical routes of lung cancer dissemination into the CSF–leptomeningeal compartment. Tumor cells may access this space through hematogenous dissemination, choroid plexus entry, perivascular or perineural spread, spinal CSF pathways, and potentially direct CSF seeding. Once established, LM may display linear or nodular growth patterns along intracranial and spinal leptomeningeal surfaces. **(B)** Ecological constraints of the CSF niche, including low protein content, limited nutrients, sparse extracellular matrix, low cellularity, reduced stromal support, pulsatile CSF flow, and barrier-regulated immune-cell entry. **(C)** Immune surveillance and barrier interfaces within the CSF–meningeal compartment. The choroid plexus epithelium, fenestrated capillaries, meningeal vessels, dura mater, and dural lymphatic system regulate immune-cell trafficking, antigen drainage, cytokine sensing, and blood–CSF exchange, thereby shaping local immune surveillance. **(D)** Compartmental comparison of peripheral blood, brain parenchymal metastasis, and CSF leptomeningeal metastasis, illustrating their distinct cellular, stromal, and fluid-phase microenvironments. These differences may contribute to the spatial and biological heterogeneity of LM, including linear and nodular disease states. CSF, cerebrospinal fluid; LM, leptomeningeal metastasis; NK cells, natural killer cells.

## 3 Tumor adaptation in CSF

### 3.1 Survival under nutrient-poor and low-protein conditions

When lung cancer cells invade the CSF, they are exposed to different conditions than in the lung, blood or brain parenchyma. There is a low protein content, limited mitogenic support and limited attachment to the extracellular matrix. Cells also need to withstand variable oxygen tensions, continuous shear forces from CSF flow and immune attacks from a small but highly specialized population of leukocytes (17–19, 31). To survive in this environment, tumor cells must be able to remain suspended, change their metabolic profile and alter the niche. The C3 model of LM offers some of the best experimental examples of this. Cancer-cell-derived C3 increased the permeability of the choroid plexus, enabling plasma proteins (including amphiregulin and other mitogens) to enter the CSF (18). Thus, the tumor cells transformed a growth-factor-depleted fluid into a more favorable milieu. They not only adapted to the host environment; they actively modified it to promote their survival.

There are also unique transcriptional characteristics of CSF-adapted tumor cells. Single-cell transcriptomic analysis of lung adenocarcinoma LM patient CTCs in CSF revealed malignant cells that were enriched with metabolic and adhesion-related programs (31). Though the cohort was small, the study provided an early cellular perspective of CSF CTCs and suggested that they could not be regarded as a simple surrogate to tumor cells in bulk tissue. Subsequent single-cell and multi-omic experiments came to the same conclusion: both malignant and immune populations can be directly profiled on CSF, and their states can differ significantly compared to those observed in corresponding blood or tissue samples (19, 22, 25, 32, 44, 45). CSF is more than just a source of diagnostic material then. It is the compartment where selective pressures and adaptive biology of LM are most apparent.

### 3.2 Metabolic rewiring and iron-dependent adaptation

Metabolic adaptation is likely to play a central role in CSF colonization, although it remains less well characterized in lung cancer LM than in other metastatic settings. Compared with plasma, CSF provides relatively little protein or lipid support. Tumor cells must therefore sustain membrane synthesis, redox homeostasis, and energy production despite limited access to extracellular nutrients (17, 18, 29). The metabolic programs detected in CSF CTCs and LM-associated malignant cells may reflect this selective pressure (19, 22, 31). Candidate processes include altered lipid handling, oxidative-stress responses, iron acquisition, and interactions with macrophage-derived trophic factors. These programs are best interpreted cautiously because many data sets remain small, but they provide a rationale for connecting CSF metabolomics, tumor-cell transcriptomics, and immune-cell phenotypes in future lung cancer LM studies.

A particularly relevant recent observation is the link between macrophage states, lipid programs, and targeted-therapy resistance. In NSCLC LM, lipid-associated macrophages were implicated in osimertinib resistance and leptomeningeal progression (22). This finding extends the idea of tumor adaptation beyond cancer-cell intrinsic programs. It suggests that CSF metabolic ecology may be co-produced by malignant cells and myeloid cells. In EGFR-mutant NSCLC, where third-generation epidermal growth factor receptor tyrosine kinase inhibitors (EGFR-TKIs) can control CNS and CSF tumor burden, residual LM may be influenced by the presence of resistant tumor clones and therapy-induced macrophage phenotypes (22, 46). Thus, in the manuscript we consider metabolic rewiring as a tumor-host property rather than just a tumor-cell property.

### 3.3 Anoikis resistance, adhesion, and leptomeningeal colonization

The CSF is a fluid environment that presents a simple challenge: tumor cells have little to adhere to. Parenchymal metastases are able to develop around blood vessels and in an already developed tissue matrix. In comparison, tumor cells in the CSF have to withstand long periods of suspension before adhering to permissive surfaces like the pia, arachnoid, nerve roots, ventricular lining, or choroid plexus-related structures (17, 31). This process must resist anoikis, apoptosis caused by detachment of the matrix, and can favor cells with epithelial-mesenchymal plasticity, stem-like characteristics, or changed expression of integrins and other adhesion molecules. In line with this model, single-cell profiling of CSF CTCs in lung adenocarcinoma revealed enrichment of pathways related to adhesion, indicating that reattachment is an important aspect of successful CSF colonization (31).

Partial EMT may be particularly relevant in the CSF. Tumor cells do not have to undergo a full epithelial-to-mesenchymal transition to grow in suspension, survive anoikis or adhere to leptomeningeal surfaces. Hybrid states might suffice, enabling cells to retain some epithelial characteristics while gaining motility, stemness, drug resistance and different integrin or cluster of CD44 signaling (47). This plasticity also has diagnostic consequences. CTC capture platforms based on fixed sets of markers may miss hybrid or highly plastic cells, but single-cell and label-flexible methods are more likely to capture the wider range of CSF-adapted tumor cell phenotypes.

Adhesion not only keeps tumor cells in place, but can also create local signaling niches. Tumor cells that adhere to meningeal or barrier surfaces can then be provided with survival signals, disrupt epithelial or endothelial junctions, and recruit macrophages and monocytes. This process is exemplified by the SPP1-MMP14 axis found in LM models. Osteopontin, which is encoded by SPP1 and secreted by cancer-cells, facilitates invasion of barriers and recruitment of macrophages (20). The fact that SPP1-positive macrophages are enriched in LM as opposed to NSCLC BrM further substantiates the role of this pathway in CNS metastasis of lung cancer (21). Colonization of the leptomeningeals can thus proceed in a chain of events: persistence in suspension, adherence to permissive surfaces, restructuring of local barriers, and recruitment of immune cells.

### 3.4 Complement activation and tumor-supportive signaling

Complement activation is among the best-supported mechanisms through which tumor cells reshape the CSF environment in LM. In the landmark C3 study, tumor-derived C3 proved necessary for efficient leptomeningeal growth in preclinical models and was also associated with human disease (18). Mechanistically, C3 activated C3a receptor signaling in the choroid plexus, disrupted the BCSFB, and allowed plasma-derived components to enter the CSF. These components included mitogens that supported cancer-cell proliferation. The study therefore recast complement as more than an immune defense pathway. In LM, it can also be co-opted by tumor cells to remodel an otherwise growth-limiting niche.

The complement axis may also intersect with immune suppression. Reviews of CSF immune landscapes across brain malignancies describe LM as a myeloid-dominant and complement-associated compartment, although disease-specific and tumor-type-specific differences remain substantial (33). Complement fragments can influence myeloid cell recruitment, vascular permeability, and inflammatory tone. However, the specific roles of C3, C3a receptor, and downstream complement components in lung cancer LM immunity remain incompletely mapped. The safest interpretation is that complement is a validated niche-adaptation mechanism and a plausible immune-modulatory circuit, but not yet a fully established therapeutic target in NSCLC LM.

### 3.5 Lineage plasticity, drug tolerance, and immune evasion

CSF adaptation occurs under therapeutic pressure, with CSF molecular studies and targeted-therapy cohorts showing resistant clones, discordant plasma-CSF evolution, and metabolic selection during treatment (48–57). Many patients with lung cancer LM have oncogene-driven disease and receive EGFR-TKIs, ALK inhibitors, or other targeted agents before or after LM diagnosis (1, 23, 46, 58, 59). These therapies can achieve clinically meaningful CNS responses, but CSF progression and acquired resistance remain common. CSF cell-free DNA profiling has shown that CSF can reveal resistance mechanisms and tumor evolution that may be absent or underrepresented in plasma (15, 16). Thus, the tumor-cell population in CSF should be viewed as a dynamic, treatment-selected reservoir. Immune escape is likely coupled to this adaptation. Tumor cells may reduce antigen presentation, express immune checkpoints, secrete myeloid-recruiting chemokines, or induce regulatory and exhausted lymphoid states (19–22, 33). The evidence for individual pathways varies, but CSF profiling studies point in a consistent direction: LM does not simply exclude immune cells. It reorganizes the local immune context toward states that fail to control tumor growth (19–22, 32, 33) (**Figure 2**).

**Figure 2:**
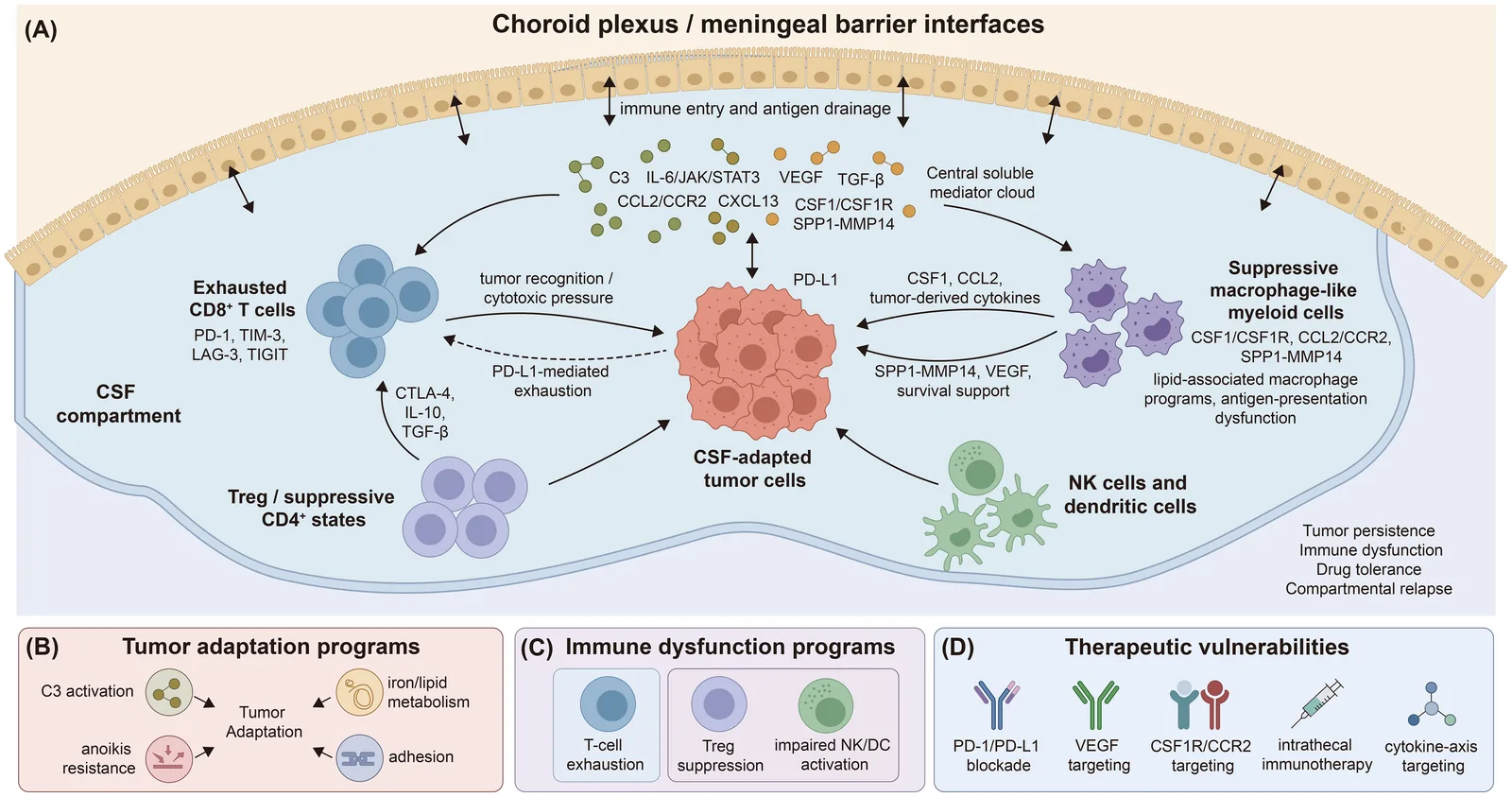
Cellular ecosystems and signaling circuits that sustain immune suppression in the cerebrospinal fluid niche. **(A)** Cellular and signaling interactions within the CSF compartment during LM. CSF-adapted tumor cells engage in direct crosstalk with exhausted CD8⁺ T cells, regulatory T-cell and suppressive CD4⁺ states, macrophage-like myeloid cells, NK cells, dendritic cells, and choroid plexus or meningeal barrier interfaces. Representative circuits include PD-1/PD-L1, CTLA-4, TIGIT, LAG-3, TIM-3, C3, IL-6/JAK/STAT3, TGF-β, VEGF, CCL2/CCR2, CXCL13, CSF1/CSF1R, MIF/CD74, and SPP1-MMP14. **(B)** Tumor adaptation programs in the CSF niche include complement activation, resistance to anoikis, iron and lipid metabolic adaptation, and adhesion-related programs that collectively support tumor survival under fluid-phase and nutrient-limited conditions. **(C)** Immune dysfunction programs are characterized by T-cell exhaustion, Treg-mediated suppression, and impaired NK-cell and dendritic-cell activation, converting immune-cell entry into ineffective or non-protective antitumor responses. **(D)** Therapeutic vulnerabilities arising from these interactions include PD-1/PD-L1 blockade, VEGF targeting, CSF1R/CCR2 targeting, intrathecal immunotherapy, and cytokine-axis targeting. Together, these mechanisms may promote tumor persistence, immune dysfunction, drug tolerance, and compartmental relapse. C3, complement component 3; CCL2, C-C motif chemokine ligand 2; CCR2, C-C motif chemokine receptor 2; CSF, cerebrospinal fluid; CSF1, colony-stimulating factor 1; CSF1R, colony-stimulating factor 1 receptor; CTLA-4, cytotoxic T-lymphocyte-associated protein 4; CXCL13, C-X-C motif chemokine ligand 13; DC, dendritic cell; IL-6, interleukin-6; IL-10, interleukin-10; JAK, Janus kinase; LAG-3, lymphocyte-activation gene 3; MIF, macrophage migration inhibitory factor; MMP14, matrix metalloproteinase 14; NK, natural killer; PD-1, programmed cell death protein 1; PD-L1, programmed death-ligand 1; SPP1, secreted phosphoprotein 1; MIF, macrophage migration inhibitory factor; STAT3, signal transducer and activator of transcription 3; TGF-β, transforming growth factor-β; TIGIT, T cell immunoreceptor with immunoglobulin and ITIM domains; TIM-3, T-cell immunoglobulin and mucin-domain containing-3; Treg, regulatory T cell; VEGF, vascular endothelial growth factor.

## 4 CSF immune ecosystems

### 4.1 T cell exhaustion and impaired cytotoxicity

The CSF immune ecosystem in LM contains fewer cells than many solid-tumor microenvironments, but its cellular composition can be informative. scRNA-seq of CSF from breast and lung cancer LM identified T cells, macrophages, malignant cells, and regulatory populations, with evidence of immune suppression compared with non-malignant controls (19). Subsequent integrative CSF profiling across leptomeningeal disease showed that CSF immune landscapes are disease-specific and can include clonally expanded T cell populations, interferon-response programs, and tissue-related immune signatures (32). These findings argue against a simple immune-empty CSF model. Instead, CSF can contain tumor-reactive or inflammatory lymphoid cells that are functionally constrained by the local ecosystem.

CD8 T cell aggregates within LM often retain intrinsic cytotoxic machinery yet cannot eliminate intrathecal tumor lesions, a well-documented paradox in tumor immunology. These T cells simultaneously express effector molecules such as granzymes and NKG7 while upregulating multiple inhibitory receptors; additionally, the CSF cytokine milieu blasts durable anti-tumor effector function. Such balance might be especially unstable in the CSF where there is a lack of antigen-presenting cells, soluble factors secreted by tumors are concentrated in a limited compartment, and CNS barriers restrict replenishment by the peripheral immune pool (29, 30, 32, 33). Whether or not there are cytotoxic T cells present is not the question at issue. Whether they are clonally expanded, survive at disease sites of interest, are metabolically healthy and get effective antigen-presentation signals.

The sequencing of TCR will play an important role in answering this question. High percentage of T cells in the CSF does not necessarily mean tumor specificity. On the other hand, a clonally expanded population can be more biologically informative than a much larger polyclonal infiltrate. Integrative CSF studies have started to correlate single-cell immune states with TCR clonality and disease-related responses (32). The next step in lung cancer LM is to identify what these expanded clones of CSF T cells are actually recognizing: lung cancer antigens, viral antigens, treatment-induced neoantigens or irrelevant inflammatory signals. In the absence of that information, it is hard to tell between failed antitumor immunity and an active but inadequate response, or between inflammation which is mostly incidental to the tumor.

### 4.2 Regulatory T cells and suppressive lymphoid states

Regulatory T cells (Tregs) are frequent residents of immunosuppressive tumor microenvironments, and CSF analysis suggests that they may also be present in LM (19, 33). Tregs can suppress multiple aspects of antitumor immunity through the expression of cytotoxic T-lymphocyte-associated protein 4 (CTLA-4), interleukin-10 (IL-10), transforming growth factor-β (TGF-β), competition for nutrients and inhibition of antigen-presenting cells. Their specific role in lung cancer LM remains to be determined. Yet, the presence of regulatory lymphoid states in the CSF provides one potential explanation for the variable clinical response to ICB, which is effective only in some patients with LM (60, 61).

Suppressive lymphoid states must be understood in the context of tumor mass and myeloid remodeling. In CSF with a high proportion of malignant cells and macrophage-like programs, Tregs may play an important role even if they are rare. They might have enhanced effects where antigen presentation and priming of T-cells is already impaired. This environment contrasts with solid tumors, where a large number of effector T cells can partially offset the regulatory influence. Thus, Tregs are considered here as part of a wider suppressive lymphoid milieu, alongside exhausted CD8 T cells, dysfunctional CD4 T cells and poor local priming (**Table 1**).

**Table 1:** Cellular and soluble components of the CSF immune ecosystem.

Component	Representative markers/readouts	Functional interpretation	Therapeutic implication
CSF-adapted tumor cells	EGFR/ALK alterations; C3; VEGF; adhesion genes; metabolic programs	Tumor survival, CSF colonization, barrier remodeling	CNS-active targeted therapy; intrathecal chemotherapy; ctDNA-guided monitoring
CSF CTCs	CTC count; EpCAM; TROP2; single-cell tumor transcriptomes	Cellular tumor burden and viable malignant compartment	CTC enumeration plus scRNA-seq for response tracking
Circulating hybrid cells	Tumor and immune/myeloid hybrid features	Possible plastic or transition states; may escape standard capture	Consider label-flexible or microfluidic enrichment
Exhausted CD8+ T cells	PD-1; TIM-3; LAG-3; TIGIT; GZMB; NKG7; TCR clonality	Cytotoxic potential with impaired effector function	ICB or intrathecal immunotherapy if clonally expanded
Regulatory T cells/suppressive CD4+ states	FOXP3; CTLA-4; IL-10; TGF-beta	Suppression of antigen presentation and cytotoxic immunity	Combination strategies that relieve immune suppression
Macrophage-like myeloid cells	SPP1; MMP14; CSF1R; CCL2/CCR2; CD44; integrins	Tumor-supportive myeloid remodeling and barrier invasion	Myeloid modulation; CSF1R/CCR2-axis targeting; combination therapy
Dendritic cells	MHC genes; antigen-presentation markers	Often numerically or functionally limited in CSF	Strategies to improve antigen presentation
NK cells	NKG7; GNLY; cytotoxicity-associated genes	Innate cytotoxic potential but often impaired activation	Potential support for cytokine or cellular immunotherapy
Cytokines/soluble mediators	IL-6; IL-10; TGF-β; VEGF; CXCL13; complement fragments	Inflammatory but often non-protective CSF signaling	Cytokine-axis targeting; anti-VEGF therapy; toxicity monitoring
CSF ctDNA/cfDNA	Driver mutations; resistance mutations; tumor fraction; MRD kinetics	Molecular tumor burden and compartmental evolution	Treatment selection; molecular response; relapse detection

CSF, cerebrospinal fluid; EGFR, epidermal growth factor receptor; ALK, anaplastic lymphoma kinase; C3, complement component 3; VEGF, vascular endothelial growth factor; ctDNA, circulating tumor DNA; CTC, circulating tumor cell; EpCAM, epithelial cell adhesion molecule; TROP2, trophoblast cell-surface antigen 2; scRNA-seq, single-cell RNA sequencing; CHC, circulating hybrid cell; PD-1, programmed cell death protein 1; TIM-3, T-cell immunoglobulin and mucin-domain containing-3; LAG-3, lymphocyte activation gene 3; TIGIT, T-cell immunoreceptor with immunoglobulin and ITIM domains; TCR, T-cell receptor; Treg, regulatory T cell; FOXP3, forkhead box P3; CTLA-4, cytotoxic T-lymphocyte-associated protein 4; IL-10, interleukin-10; TGF-β, transforming growth factor-β; SPP1, secreted phosphoprotein 1; MMP14, matrix metalloproteinase 14; CSF1R, colony-stimulating factor 1 receptor; CCL2, C-C motif chemokine ligand 2; CCR2, C-C chemokine receptor type 2; CD44, cluster of differentiation 44; MHC, major histocompatibility complex; NK, natural killer; NKG7, natural killer cell granule protein 7; GNLY, granulysin; CXCL13, C-X-C motif chemokine ligand 13; cfDNA, cell-free DNA; MRD, molecular residual disease.

### 4.3 Myeloid remodeling and macrophage-like programs

Myeloid remodeling is one of the more consistently observed immune features across available LM and CSF studies, although the direct lung cancer LM evidence is still limited. The initial CSF scRNA-seq study of breast and lung cancer LM reported macrophages with M2-like transcriptional profiles and a general trend toward immunosuppression (19). A later review of CSF immune landscapes across brain malignancies synthesized evidence that LM is often macrophage-rich and may involve complement-associated immune reprogramming, but this source should be read as a review-level synthesis rather than primary lung cancer LM evidence (33). Primary comparative evidence from NSCLC showed that LM had fewer lymphocytes and enrichment of SPP1-positive macrophages, whereas BrM retained more organized immune activity, including CXCL9-positive macrophages and CXCL13-positive CD4 T cells (21). Additional NSCLC data link lipid-associated macrophage programs to osimertinib resistance and LM development (22).

These myeloid states are not likely to be bystanders. Macrophages can actively promote LM by secreting growth factors, remodeling the extracellular matrix, compromising barrier integrity, changing antigen presentation and inhibiting T-cell responses. Mechanistic evidence is provided by the study of macrophages derived from the dura, which demonstrated that LM-associated macrophages are able to invade the CSF and contribute to the creation of an immunosuppressive milieu characterized by T-cell exhaustion and functional inactivation (20). The SPP1-MMP14 axis is especially informative since it connects tumor-derived signals with macrophage recruitment, matrix remodeling, and invasion of barriers (20). This model aligns with the enrichment of SPP1-positive macrophages in NSCLC LM and confirms the opinion that myeloid reprogramming is a key characteristic of the LM ecosystem (21).

A binary M1/M2 paradigm fails to reflect the complexity of macrophage diversity. Border-associated macrophages, monocyte-derived macrophages, lipid-associated macrophages, SPP1-positive macrophage-like cells and treatment-experienced populations can be found in the CSF and meninges (20–22, 30). These may vary in their cellular origin, antigen-presenting function, lipid metabolism, matrix-remodeling and drug sensitivity. The population of lipid-associated macrophages found in NSCLC LM is an example of this diversity, with a connection between altered macrophage lipid metabolism and osimertinib resistance (22). Thus, CSF myeloid cells should be viewed as a spectrum of states rather than discrete entities, with specific functions in immune suppression, adaptation to the tumor environment, and drug tolerance.

### 4.4 Natural killer cells, dendritic cells, and defective immune activation

Natural killer (NK) cells and dendritic cells (DCs) are less studied in lung cancer LM than T cells and macrophages, but play a pivotal role in explaining the failure of immune activation. NK cells are able to kill tumor cells without prior antigen recognition, whereas DCs facilitate effective T-cell priming and antigen presentation. In physiological conditions, the immune surveillance at CNS borders relies on specialized trafficking pathways of leukocytes and antigen processing across the choroid plexus, meninges, and dural lymphatic interfaces (26–30). These functions can be too weak in LM, diverted not towards antitumor activity, or actively inhibited by signals sent by tumor and myeloid cells.

CSF profiling has revealed a diverse mixture of lymphoid and myeloid populations. Studies of LM arising from lung cancer, melanoma, breast cancer, and glioma provide further context for interpreting cytotoxic, interferon-responsive, and myeloid-associated immune states (19, 32, 33, 62–71). A scarcity of conventional DCs could weaken local T-cell priming, while impaired NK-cell activity might allow tumor cells with limited antigen presentation to escape immune clearance. These mechanisms are plausible, but the evidence remains indirect. Paired scRNA-seq, TCR sequencing, protein profiling, and functional assays will be needed to test them. NK cells and DCs are therefore best viewed as understudied components of CSF immunity rather than established drivers of lung cancer LM.

### 4.5 Inflammatory but non-protective CSF immune states

Inflammation in the CSF should not be taken as evidence of protective immunity. Patients with LM may have elevated cytokine levels, barrier disruption, increased CSF protein, and greater immune-cell recruitment, yet these changes can arise from tumor-promoting inflammation, tissue damage, or treatment-related effects rather than effective antitumor activity (2, 3, 25, 33, 44, 45). This distinction matters when interpreting CSF biomarkers. A cytokine-rich profile may reflect immune activation, but it can just as readily signal increased vascular permeability, macrophage influx, or neurological toxicity.

A functional classification might be the most informative way to think about immune states in the CSF. An inflamed state would be defined by clonally expanded cytotoxic T cells and interferon-response programs. A myeloid-suppressive state, on the other hand, could include SPP1-positive or lipid-associated macrophage programs, low antigen presentation and exhausted T cells. In a tumor-dominated cold state, there are many malignant cells but few effective immune cells. Cytokine-high inflammatory states are more complex: they may be caused by barrier damage, vascular endothelial growth factor (VEGF) signaling, interleukin-6 (IL-6)-driven inflammation, or drug treatment. These are not definitive categories, but they offer a convenient bridge from single-cell data to therapeutic approaches (**Table 1**).

## 5 Tumor-immune niche crosstalk

### 5.1 Immune checkpoint circuits

Tumor-immune crosstalk in CSF is likely mediated by a limited but potent set of cell-surface and soluble interactions, but the evidence for immunotherapy in LM should be attributed by tumor type and study design. For NSCLC, the literature includes reviews of brain and leptomeningeal metastases, small clinical series, and case-based evidence, indicating biological plausibility but limited prospective data (62, 63, 72, 76). Mixed solid-tumor LM studies and trials provide additional support for intrathecal or combined immune strategies, but their conclusions cannot be assumed to apply uniformly to lung cancer LM (24, 73, 77). Melanoma LMD studies and reviews support the broader concept that leptomeningeal immune states can influence treatment response, whereas breast cancer LM and breast BrM/LMD studies mainly serve as cross-cancer context (64–67, 69–71). Thus, programmed cell death protein 1 (PD-1) and programmed death-ligand 1 (PD-L1), CTLA-4, lymphocyte activation gene 3 (LAG-3), T cell immunoreceptor with immunoglobulin and immunoreceptor tyrosine-based inhibitory motif domains (TIGIT), and T cell immunoglobulin and mucin-domain containing-3 (TIM-3) are plausible mediators of T cell dysfunction in LM, but the evidence base is uneven. Checkpoint expression in CSF should therefore be interpreted as a hypothesis-generating signal rather than proof of checkpoint dependence.

A more useful crosstalk model asks which CSF immune states are checkpoint-reversible and which are dominated by tumor burden or myeloid suppression. If CSF contains clonally expanded T cells with retained cytotoxic machinery, PD-1 or CTLA-4 blockade may restore some function, but this inference is supported unevenly across NSCLC, melanoma, breast cancer, and mixed solid-tumor LM studies (62–67, 69–73, 76, 77). If CSF is dominated by malignant cells, suppressive macrophages, low antigen presentation, and limited T cell entry, checkpoint blockade alone may have little substrate to activate; this model is most directly supported by CSF studies in breast/lung LM, NSCLC LM macrophage studies, LUAD-LM CSF profiling, and broader CSF immune-landscape syntheses (19, 21, 22, 32, 33, 58). This separation of direct lung cancer LM evidence from BrM and cross-cancer literature better explains why immunotherapy responses in LM are heterogeneous despite a strong rationale for local immune modulation.

Local delivery may alter this balance. Intrathecal immune checkpoint inhibition and related immune strategies seek to raise drug exposure within the CSF while bypassing some of the barriers that limit systemic trafficking (23, 24). However, local delivery does not solve every biological problem. A checkpoint antibody in CSF still requires responsive T cells, sufficient antigen presentation, and a microenvironment not dominated by irreversible myeloid suppression or overwhelming tumor burden. These checkpoint circuits are depicted in **Figure 2** as conditional interactions whose effects depend on the broader ecosystem.

### 5.2 Chemokine axes shaping immune-cell recruitment

Chemokines are likely to determine which immune cells enter and persist in the CSF compartment. Candidate axes include C-C motif chemokine ligand 2-C-C chemokine receptor type 2 (CCL2-CCR2) for monocyte recruitment, C-X-C motif chemokine ligand 12-C-X-C chemokine receptor type 4 (CXCL12-CXCR4) for tumor-cell retention and immune-cell trafficking, C-X-C motif chemokine ligand 8-C-X-C chemokine receptor type 2 (CXCL8-CXCR2) for neutrophil-associated inflammation, and CXCL9/C-X-C motif chemokine ligand 10-C-X-C chemokine receptor type 3 (CXCL10-CXCR3) for effector T cell recruitment. Not all of these pathways have been functionally validated in lung cancer LM, but they are consistent with broader CNS border immunology and tumor microenvironment biology (21, 26–30, 33). The NSCLC BrM-LM comparison is particularly informative because BrMs showed more CXCL9-positive macrophages and CXCL13-positive CD4 T cells than LM, suggesting that chemokine architecture may distinguish immune-organized BrM from immune-suppressed LM (21).

Chemokine imbalance can produce an inflammatory but ineffective niche. Recruitment of monocytes or neutrophils without efficient antigen presentation may increase tissue injury, barrier permeability, and tumor-promoting cytokines. Conversely, insufficient CXCR3-associated effector recruitment or CXCL13-associated lymphoid organization may limit local antitumor immunity. These hypotheses now need to be tested directly in lung cancer LM, ideally through paired analyses of CSF and tissue. In **Figure 2**, chemokines are therefore shown as signals that shape whether CSF becomes T cell inflamed, myeloid suppressive, or cytokine-high, rather than as simple recruitment arrows.

### 5.3 Myeloid-tumor feedback loops

Bidirectional communication between cancer cells and myeloid cells could be a key factor in LM progression. Cancer-secreted factors attract macrophages and program their function. These macrophages can then support tumor growth by providing trophic factors, controlling lipid metabolism, extracellular matrix remodeling and suppressing immune responses (19–22, 33). The SPP1-MMP14 interaction is one of the most obvious examples to date. In experimental LM, MMP14-expressing cancer cells attracted dura-derived LM-associated macrophages. These required MMP14 to breach the CSF barrier and created a T cell-suppressive environment (20). As predicted by this model, NSCLC LM had more SPP1-positive macrophages than BrM from the parenchyma (21).

Another potential mechanism for maintaining macrophage populations and regulating myeloid states is colony-stimulating factor 1 (CSF1)-CSF1 receptor (CSF1R) signaling, but this has yet to be confirmed in lung cancer LM. Other candidate interactions such as macrophage migration inhibitory factor (MIF)-CD74, integrin-SPP1 and CD44-SPP1 may regulate myeloid-cell retention, tumor cell adhesion or immunosuppressive signals, respectively. But ligand-receptor predictions do not establish that these interactions are necessarily active. Despite this caveat, results from CSF scRNA-seq, direct comparisons of NSCLC BrM-LM, and dura-derived macrophage models point to the same overall conclusion: bidirectional communication between macrophages and tumor cells is a key aspect of LM biology (19–22).

These circuits are best introduced through an explicit evidence hierarchy. The strongest mechanistic support comes from tumor-derived C3 adaptation of CSF and SPP1-MMP14-mediated recruitment of LM-associated macrophages in experimental models (18, 20). The strongest human lung cancer support comes from NSCLC or LUAD-LM studies showing SPP1-positive macrophage enrichment, CSF-CTC transcriptional adaptation, lipid-associated macrophage programs, and TROP2-positive malignant cells (21, 22, 31, 58). Mixed breast/lung LM and pan-CNS CSF studies support the broader pattern of macrophage-dominant or disease-specific immune remodeling but should be interpreted as supportive rather than lung cancer-specific (19, 32, 33). Candidate ligand-receptor axes, including CSF1-CSF1R, CCL2-CCR2, MIF-CD74, SPP1-integrin, and SPP1-CD44, should therefore be presented as testable pathways rather than established functional mechanisms in lung cancer LM.

### 5.4 Cytokine, VEGF, and complement-mediated suppression

Another dimension to this crosstalk is soluble mediators. IL-6, TGF-beta, VEGF, fragments of the complement and inflammatory factors secreted by macrophages are able to modify the permeability of barriers, immune-cell recruitment, T-cell activity, and tumor-cell survival (18, 20, 22, 25, 33, 44, 45). VEGF can be especially applicable in LM where alterations in vascular permeability and CSF protein content may affect symptoms, drug distribution, and leukocyte trafficking. The inflammation associated with IL-6 can also facilitate myeloid activation and treatment resistance but its role in lung cancer LM is not clear.

Complement deserves special consideration since its contribution to LM has been proven experimentally. C3 produced by tumor cells breaks the choroid plexus barrier and enables plasma mitogens to access CSF, directly associating malignant-cell activity with a host barrier response (18). C3 can also affect myeloid recruitment and the inflammatory milieu in general but these immune effects have not yet been characterized in NSCLC LM. The evidence thus substantiates C3 as a known mechanism of CSF niche adaptation, whereas its immunomodulatory role downstream is a possibility that has not yet been proven.

### 5.5 Integrated signaling networks as therapeutic targets

The above signaling circuits are unlikely to act independently, and the review’s synthesis is that they can be grouped into four practical axes rather than a long catalogue of targets. First, a malignant-cell adaptation axis includes CSF-CTCs, metabolic stress programs, adhesion, complement C3, lineage plasticity, and CSF ctDNA-defined molecular evolution (15–18, 31, 58). Second, a myeloid-suppression axis includes SPP1-positive macrophages, MMP14-dependent border invasion, lipid-associated macrophages, CSF1/CSF1R, CCL2/CCR2, MIF/CD74, CD44, and integrin-associated signaling (20–22). Third, a lymphoid-dysfunction axis includes exhausted CD8 T cells, regulatory CD4 or Treg-like states, PD-1/PD-L1, CTLA-4, TIGIT, LAG-3, TIM-3, and TCR clonality (19, 21, 32, 58). Fourth, a barrier-inflammatory axis includes IL-6/JAK/STAT3, VEGF, TGF-beta, complement, cytokine-high inflammation, and blood-CSF barrier dysfunction (18, 20, 22–25). This axis-based framework is more specific than the general phrase ‘tumor-immune ecosystem’ and can be tested with paired CSF and blood profiling.

The main interpretive point is therefore more specific than stating that lung cancer LM is a CSF-confined tumor-immune ecosystem. The available evidence suggests that lung cancer LM may be driven by the interaction of a tumor-burden module, a macrophage-like myeloid module, a dysfunctional lymphoid module, and a barrier-inflammatory module. These modules are not equally proven: CSF ctDNA/CTC detection and selected LUAD-LM scRNA-seq findings provide relatively direct clinical evidence, C3 and SPP1-MMP14 provide experimental mechanistic evidence, and CSF1/CSF1R, CCL2/CCR2, MIF/CD74, checkpoint, VEGF, and IL-6/JAK/STAT3 axes remain candidate therapeutic or monitoring hypotheses. This framing provides the foundation for the therapeutic sections without implying that tumor-directed therapy or systemic biomarkers are sufficient as single readouts. Successful future management may require concurrent reduction in CSF tumor burden, recovery or preservation of functional lymphoid immunity, interruption of macrophage-tumor feedback, and longitudinal monitoring of compartmental disease progression in CSF (**Figure 2**).

## 6 Therapy-induced remodeling

### 6.1 Immune checkpoint blockade in LM

The treatment of lung cancer LM can be more accurately viewed as therapy-induced remodeling, rather than a simple issue of whether a drug is able to access the CSF. A particular treatment can reduce the tumor burden in the CSF, alter the composition of malignant clones, alter permeability of barriers, and alter the state of T cells or myeloid cells (74, 75). ICB exhibits such complexity. The blockade of PD-1, PD-L1, and CTLA-4 may result in long-lasting responses in extracranial NSCLC and in some BrM, yet patients with LM have been historically underrepresented in clinical studies (3, 24, 72). Clinical data available indicate that there are patients with LM who may benefit out of ICB. Responses, however, are very variable and might be determined by the presence of an immune substrate in the CSF which can still be reactivated (24, 72, 73, 76, 77).

To translate to the clinic, CSF exposure is not enough. ICB will be more effective if CSF T cells are reversible by checkpoint blockade. Checkpoint inhibition can reinvigorate antitumor activity in a compartment containing clonally expanded cytotoxic T cells and sufficient antigen presentation. With a crowded compartment of malignant cells, SPP1-positive macrophages, weak dendritic cell stimulation and exhausted or sparse T cells, ICB may have little to go on (19–22, 24, 32, 33). This has led to interest in intrathecal nivolumab, and intrathecal nivolumab plus interleukin-2 (IL-2): local administration can boost CSF concentrations while activating immunity locally (76, 77). Preliminary studies and case reports are promising but preliminary. Currently they support feasibility and biological plausibility rather than an established standard for NSCLC LM.

A CSF-immune framework also makes biomarker development central to immunotherapy in LM. Peripheral PD-L1 expression, tumor mutational burden, and blood immune markers may say little about local T cell dysfunction or myeloid suppression within the CSF. CSF proteomic data have identified adhesion G protein-coupled receptor G1 (ADGRG1) as a candidate biomarker of response to intrathecal immune checkpoint inhibitors, suggesting that compartmental biomarkers may outperform systemic markers in selected patients (23). Future trials should pair CSF scRNA-seq, TCR sequencing, protein profiling, and CSF tumor-derived DNA so that investigators can distinguish immune activation and tumor clearance from inflammatory toxicity.

### 6.2 Targeted therapies and compartmental tumor control

Targeted therapy remains a core part of lung cancer LM treatment because many patients have oncogene-driven NSCLC. EGFR-TKIs, ALK inhibitors, and other CNS-active targeted agents can reduce tumor burden and improve neurological symptoms in selected patients (1, 46, 59, 78). Their effects, however, extend beyond direct tumor cytotoxicity. When targeted agents reduce malignant cells in CSF, they may also change antigen release, cytokine levels, immune-cell proportions, and macrophage programs. The same pressure can also select resistant clones and treatment-adapted host states, including lipid-associated macrophage programs linked to osimertinib resistance (22).

A number of recent papers demonstrate the evolution of the role of targeted treatment in LM. High-dose furmonertinib has demonstrated activity in EGFR-mutant NSCLC with LM, and combinations of furmonertinib are now being tested in larger cohorts in real-world settings (78–84). Lazertinib and pemetrexed has also been evaluated in EGFR-mutant NSCLC LM, as part of a general trend toward combining CNS-penetrant targeted therapy with cytotoxic or local therapy (85–90). These results should be interpreted with caution, as many studies are single-arm, retrospective or have an enriched population of patients with targetable drivers. Yet they suggest a pragmatic approach to treatment: targeted therapy may offer rapid control of compartmental disease, but long-term benefit will likely require understanding CSF molecular progression and combining tumor-targeted treatment with approaches that target immune and myeloid remodeling.

### 6.3 Intrathecal chemotherapy and local immune perturbation

Intrathecal chemotherapy is still a valuable treatment for LM as it allows the delivery of cytotoxic drugs to CSF tumor cells, particularly in the setting of low systemic drug exposure (2, 3, 91, 92). Intrathecal pemetrexed has been of particular interest in lung cancer LM. It can be delivered by lumbar puncture or an Ommaya reservoir and has demonstrated clinical activity in selected NSCLC patients (91, 92). The most common explanation is pharmacologic: intrathecal administration overcomes systemic barriers and increases the concentration of drug in the CSF. An immune-ecological perspective provides a second reason. Lowering the number of malignant cells, intrathecal chemotherapy may also increase the presence of tumor antigens, alter cytokine levels, and alter the ratio of tumor cells to immune cells.

These effects may be beneficial or detrimental to the patient, depending on the CSF environment. Killing of tumor cells can create an opportunity for immune recognition but can also lead to inflammation, neurotoxicity or disruption of the blood-brain barrier. The degree of remodeling probably relates to the initial CSF tumor load, obstruction of CSF flow, the type of immune cells and the presence of targeted therapy or radiotherapy. Reviews of intrathecal chemotherapy, Ommaya reservoir chemotherapy, and multimodal local therapies reveal an increasing amount of data, but there is still a lack of uniformity among regimens, patients, and response definitions (80–84, 86–92). Randomized trials such as intrathecal pemetrexed plus bevacizumab in NSCLC LM may help clarify whether local chemotherapy can be combined with vascular and immune-modulating approaches (93).

### 6.4 Radiotherapy, antigen release, and barrier injury

Radiotherapy is still valuable for bulky nodular disease, symptomatic sites, craniospinal disease in certain circumstances and underlying parenchymal BrM (2, 3, 72, 94). But its effects on the immune system are two-fold. Radiation can destroy tumor cells, present antigens, stimulate inflammatory signals, and may synergize with ICB. It can also damage CNS tissue, disrupt barriers, exacerbate swelling and trigger myeloid repair responses that could dampen long-term immunity. In LM, these factors are more important because the disease follows CSF pathways, craniospinal surfaces and vulnerable neurology.

The phase IB trial of avelumab and whole-brain radiotherapy in patients with leptomeningeal disease from solid tumors demonstrates how researchers are starting to combine radiation with immune stimulation and molecular surveillance (73). The data is still preliminary, but the question is worth posing: can local radiation damage not only alleviate symptoms but also help induce effective antitumor immunity? In NSCLC LM, this will depend heavily on patient selection. Diffuse linear LM, nodular LM, obstructed CSF flow, symptomatic BrM, and poor performance status do not call for the same radiation strategy. Future protocols should therefore report more than survival and imaging response. They should also include CSF biomarkers that help separate antigen-releasing immune activation from non-protective inflammation.

### 6.5 Anti-VEGF and emerging intrathecal immunotherapies

VEGF is relevant to LM because barrier permeability, CSF protein elevation, edema, and vascular injury can affect symptoms and drug distribution. Anti-VEGF therapy, including bevacizumab or ramucirumab-based combinations, may reduce permeability and may indirectly change immune-cell trafficking or myeloid suppression (93, 95). Evidence in NSCLC LM remains preliminary, but combinations such as furmonertinib plus bevacizumab and intrathecal pemetrexed plus bevacizumab reflect growing interest in targeting both tumor cells and the vascular-CSF interface (93, 95).

Emerging intrathecal immunotherapies build on the same local-treatment logic. Intrathecal nivolumab, intrathecal cytokine combinations, and intrathecal or intracompartmental cellular therapies all try to bring immune activity directly into the CSF (76, 77, 96, 97). B7 homolog 3 (B7-H3)-targeted allogeneic chimeric antigen receptor (CAR) gamma-delta T cells have already entered early-phase testing for LM from solid tumors. Initial evidence suggests persistence of CSF, interferon-gamma-related immune remodeling, and a tolerable yet significant risk of neurotoxicity (96, 98). Other approaches such as bispecific antibodies, oncolytic viruses and engineered cellular vehicles are less advanced in lung cancer LM. Nevertheless, they are applicable since they combine local tumor killing with the activation of immunity (61, 99). These strategies will require a cautious consideration regarding exposure to corticosteroids, immune effector cell-associated neurotoxicity syndrome, cytokine release, hydrocephalus, and pre-existing neurological reserve. In this regard, they put LM in the context of a compartmental immune disease, not just a pharmacologic sanctuary.

## 7 CSF multi-omics

### 7.1 Single-cell RNA sequencing of CSF ecosystems

CSF multi-omics may become a translational bridge between mechanism and therapy, but current evidence should be separated by disease context. In lung cancer LM, scRNA-seq and CSF-CTC studies have shown malignant-cell programs related to adhesion, metabolism, metastatic competence, and CSF adaptation (31, 58). Mixed breast and lung cancer LM profiling supports the presence of malignant and immune-cell compartments, including regulatory and macrophage-skewed states, but its small cross-cancer sample composition should be acknowledged (19). Broader CSF profiling across CNS lymphoma, BrM, glioblastoma, and LMD identifies disease-specific immune responses and supports the general value of CSF immune monitoring, but it is not lung cancer LM-specific evidence (32). Breast-cancer LM multimodal CSF profiling provides useful methodological context for serial CTC, single-cell, and proteomic assessment rather than direct support for lung cancer LM biology (44). The TROP2-positive malignant-cell finding in LUAD-LM is best attributed specifically to the LUAD-LM CSF study that evaluated its diagnostic value (58).

For clinical translation, scRNA-seq should be used to classify functional states rather than to create increasingly long cell lists. A sample may be T cell inflamed, myeloid suppressive, tumor dominant, or cytokine-high. These states could guide therapy: T cell inflamed CSF may support ICB or intrathecal immunotherapy, whereas myeloid suppressive CSF may require macrophage-directed combinations. This approach is likely to require harmonized processing, sufficient cell recovery, viable-cell thresholds, and careful distinction between true biological absence and technical dropout. Cryopreservation protocols for fragile CSF cells show that multicenter scRNA-seq and paired TCR/B cell receptor profiling can be feasible when collection and freezing procedures are standardized (100). It will also require integration with CSF tumor-derived DNA, because a low-cell sample may still contain substantial molecular evidence of disease.

### 7.2 TCR profiling and local antigen-driven immunity

To interpret CSF T cells, TCR profiling is required to do so with any degree of confidence. An elevated number of T cells is not a sufficient indicator of antitumor immunity and an exhausted phenotype is not necessarily tumor specific. TCR sequencing may reveal the presence of clonally expanded CSF T cells, whether these clones survive therapy, and whether their expansion correlates with tumor control (32). TCR profiling can be used in conjunction with scRNA-seq to distinguish between bystander inflammation and local antigen-driven responses. This difference is important in LM as CSF inflammation can be indicative of tumor progression, treatment response, infection or barrier damage.

Future studies of immunotherapy should obtain TCR profiles pre-treatment, during early response and again at progression. Increased cytotoxic clones along with decreased tumor-derived DNA in the CSF of responding patients would confirm immune control. Failure to progress despite clonal expansion would suggest loss of antigen or myeloid suppression or ongoing T cell dysfunction. In the absence of clonal expansion, checkpoint blockade or local cytokine therapy may require combination with strategies that enhance antigen presentation. In this context, TCR information could transform descriptive CSF immunology into immune monitoring for treatment guidance.

### 7.3 CSF ctDNA and tumor evolution

One of the more clinically developed biomarkers to lung cancer LM is CSF ctDNA. It can aid in diagnosis, estimate tumor burden, identify driver mutations and resistance alterations, and compartmental evolution which may not be detected by plasma testing (15, 16, 101, 102). In lung cancer LM suspected, the measurement of CSF tumor DNA can enhance molecular detection over cytology alone (15). CSF cell-free DNA profiling has also demonstrated clinical utility and predictive value in NSCLC LM, which justifies its application in treatment selection and disease monitoring (16). In the future, CSF ctDNA is expected to be used as a genotyping tool but also as longitudinal molecular residual disease (MRD) readout of compartmental disease activity.

The value of CSF ctDNA extends beyond genomics, but direct LM evidence and cross-sample methodological analogies should be distinguished. In NSCLC and solid-tumor LM, systematic reviews and meta-analyses support CSF ctDNA as a diagnostic, prognostic, and disease-activity biomarker, while also emphasizing the need for standardized sampling, assays, and longitudinal thresholds (101, 102). When combined with immune profiling, CSF ctDNA may help distinguish tumor-directed response from inflammatory change. A decline in CSF ctDNA accompanied by increased T cell activation may point to effective immune-tumor control, whereas rising ctDNA despite cytokine elevation may suggest inflammation without tumor protection. Lessons from plasma MRD and tumor-naive ctDNA studies are methodologically useful, especially for methylation, fragmentomics, copy-number changes, and mutation-integrated assays, but refs 103–104 are plasma or colorectal/pan-cancer studies and should be treated as cross-sample analogies rather than direct LM CSF evidence (103, 104). In LM, a false-negative CSF ctDNA result may reflect low tumor shedding, sampling away from nodular disease, limited CSF volume, prior therapy, or the assay limit of detection (LOD). CSF ctDNA therefore provides a molecular backbone for CSF-guided care, while scRNA-seq, TCR profiling, CTCs, cytokines, and proteomics help interpret the immune and cellular ecology around it.

In practice, CSF ctDNA platforms should be judged against three questions. Can the assay detect the relevant lung cancer alteration or tumor fraction at the low input levels typical of CSF? Can it separate molecular response from sampling variability and treatment-induced inflammation? Can it be combined with immune readouts without using up the limited CSF volume available from symptomatic patients? These issues have direct clinical consequences. A negative CSF ctDNA result should not exclude LM by itself when MRI, cytology, symptoms, or immune-state data still point to active disease.

### 7.4 CSF CTCs, cytokines, proteomics, and metabolomics

CSF CTC enumeration and characterization can provide cellular data that ctDNA cannot, such as tumor-cell burden, cytologic-molecular correlation, single-cell phenotypes, and longitudinal response patterns (105–114). The number of CTCs provides prognostic information in LM with epithelial tumors and can be used to indicate treatment response when monitored over time (45). Single-cell transcriptomics of CSF CTCs is also capable of revealing malignant programs associated with adhesion, metabolism, lineage state, and therapeutic vulnerability (31). Here technical choices are important. Epithelial cell adhesion molecule (EpCAM)-based rare-cell capture can work well for epithelial tumors. Label-free or microfluidic approaches may recover a broader range of cells, including those with partial epithelial-mesenchymal transition, low epithelial marker expression, or hybrid tumor-immune phenotypes (115, 116). A recent CSF microfluidic study detected both CTCs and circulating hybrid cells (CHCs) in LM, illustrating how enrichment strategy can shape the cellular compartment that is recovered (115). CTCs may still underrepresent adherent nodular disease, depend on sampling site and CSF flow, and be difficult to recover in low-volume specimens. For this reason, CTC analysis should be integrated with ctDNA rather than treated as a replacement.

Proteomics, cytokine profiling, extracellular vesicle analysis, and metabolomics can capture non-cellular components of the CSF ecosystem (23, 25, 44, 117, 118). Proteomic studies may identify predictors of immunotherapy response, such as ADGRG1 in intrathecal ICB, while cytokines can indicate inflammatory or barrier-injury states (23). Extracellular vesicle-based liquid biopsy is an emerging area with potential to reveal tumor and immune communication, but current evidence remains early (117). Metabolomics may be particularly relevant for LM because CSF is nutrient constrained and tumor-host metabolic adaptation appears important (22). Together, these platforms can define the soluble layer of the CSF ecosystem.

### 7.5 Longitudinal CSF monitoring during treatment

The strongest use of CSF multi-omics will be longitudinal monitoring. LM is dynamic: tumor burden, resistance mutations, immune-cell states, cytokines, and barrier function can change rapidly after targeted therapy, intrathecal chemotherapy, radiotherapy, or immunotherapy (15, 16, 119). Serial CSF monitoring through an Ommaya reservoir has been proposed as a way to follow treatment efficacy and prognosis in lung cancer LM (119). This approach can reduce repeated lumbar puncture burden and allows more consistent sampling, although reservoir placement is not suitable for all patients.

Clinical decisions should be the basis of longitudinal monitoring. CSF profiling can be used to confirm LM and characterize driver mutations and local immune state at diagnosis. It can differentiate between molecular response and radiographic uncertainty during therapy. At progression, it can reveal resistance mechanisms and determine whether failure is tumor dominant, immune suppressive, or inflammatory. In **Figure 3**, depicts a closed-loop clinical workflow including standardized CSF sampling, multi-omic profiling, immune subtyping, personalized treatment selection, and longitudinal adaptive monitoring.

**Figure 3:**
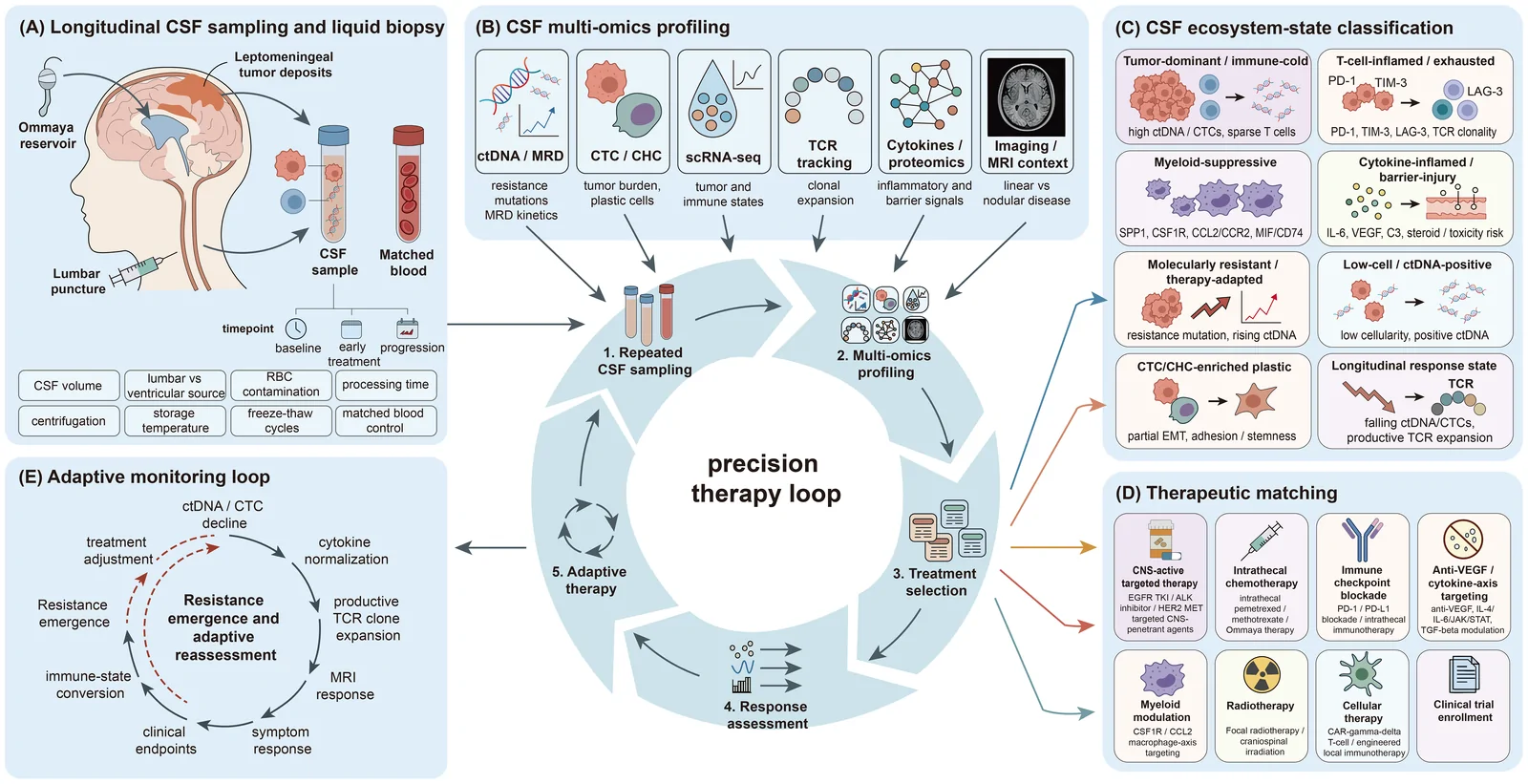
Cerebrospinal fluid profiling-guided treatment decision model for lung cancer leptomeningeal metastasis. **(A)** Longitudinal CSF sampling and liquid biopsy through lumbar puncture or Ommaya reservoir, with paired blood collection, enable serial assessment across baseline, early treatment, and progression. Standardization of CSF volume, sampling source, RBC contamination, processing time, centrifugation, storage conditions, freeze–thaw cycles, and matched blood controls is essential for reliable longitudinal analysis. **(B)** CSF multi-omics profiling integrates ctDNA or MRD kinetics, CTCs and CHCs, scRNA-seq, TCR tracking, cytokine and proteomic profiling, and imaging context to characterize tumor burden, resistance mutations, immune states, clonal dynamics, inflammatory signals, and radiographic disease patterns. **(C)** Integrated readouts classify CSF ecosystems into actionable states, including tumor-dominant immune-cold, T-cell-inflamed exhausted, myeloid-suppressive, cytokine-inflamed barrier-injury, molecularly resistant or therapy-adapted, low-cell ctDNA-positive, CTC/CHC-enriched plastic, and longitudinal response states. **(D)** Ecosystem states are linked to rational therapeutic options, including CNS-active targeted therapy, intrathecal chemotherapy, immune checkpoint blockade, anti-VEGF or cytokine-axis targeting, myeloid modulation, radiotherapy, cellular therapy, and clinical-trial enrollment. **(E)** Serial reassessment closes the precision-therapy loop by integrating ctDNA or CTC kinetics, cytokine normalization, productive TCR-clone expansion, MRI and symptom responses, clinical endpoints, immune-state conversion, and resistance emergence to guide adaptive treatment adjustment. ALK, anaplastic lymphoma kinase; C3, complement component 3; CAR, chimeric antigen receptor; CCL2, C-C motif chemokine ligand 2; CCR2, C-C motif chemokine receptor 2; CHC, circulating hybrid cell; CNS, central nervous system; CSF, cerebrospinal fluid; CSF1R, colony-stimulating factor 1 receptor; CTC, circulating tumor cell; ctDNA, circulating tumor DNA; EGFR, epidermal growth factor receptor; EMT, epithelial-to-mesenchymal transition; HER2, human epidermal growth factor receptor 2; IL-4, interleukin-4; IL-6, interleukin-6; JAK, Janus kinase; MET, mesenchymal-epithelial transition factor; MIF, macrophage migration inhibitory factor; MRD, molecular residual disease; MRI, magnetic resonance imaging; PD-1, programmed cell death protein 1; PD-L1, programmed death-ligand 1; RBC, red blood cell; scRNA-seq, single-cell RNA sequencing; SPP1, secreted phosphoprotein 1; STAT, signal transducer and activator of transcription; TCR, T-cell receptor; TGF-β, transforming growth factor-β; TKI, tyrosine kinase inhibitor; VEGF, vascular endothelial growth factor.

### 7.6 Standardizing CSF immune profiling for clinical translation

The most immediate barrier to CSF-guided care is pre-analytical reproducibility as much as assay cost or sequencing depth. CSF volume, sampling site, time to processing, temperature, centrifugation, red blood cell contamination, freeze-thaw history, storage tube, and whether cells or supernatant are prioritized can all alter downstream readouts. Proteomic studies show that processing delay, storage temperature, repeated freeze-thaw cycles, and blood contamination can bias CSF measurements, even when CSF is more stable than plasma under some conditions (120). For scRNA-seq and TCR profiling, the relevant variables include viable-cell recovery, doublet rate, ambient RNA, immune-cell dropout, and whether fragile cells are profiled fresh or cryopreserved (100).

An effective CSF profiling protocol must record the information that can frequently make or break a result: precise volume of CSF, lumbar or ventricular origin, opening pressure (where available), red blood cell count, nucleated cell count, processing time, centrifugation parameters, storage temperature and freeze-thaw cycles. Whenever feasible, matched blood is to be collected to detect systemic contamination, common TCR clones, clonal hematopoiesis, and plasma-derived proteins. There should also be restraint in multi-omic integration. Batch correction comes in handy, but too aggressive correction may wipe off actual differences in disease states, particularly with small cohorts. The aim of the translational is not to produce the biggest dataset possible. It is to construct a lean, reproducible panel: ctDNA or MRD kinetics of tumor burden, CTC or circulating hybrid-cell recovery of cellular disease, scRNA-seq or targeted gene/protein panels of immune state, TCR clonality of antigen-driven immunity, and cytokine or proteomic markers of barrier injury and inflammation. These readouts give the measurement backbone of the taxonomy of immune-states summarized in **Table 2**.

**Table 2:** Actionable CSF immune-state taxonomy, biomarkers and therapeutic implications.

CSF immune state	Candidate biomarkers	Technical caveats	Therapeutic implication
Tumor-dominant immune-cold	High CSF ctDNA or CTCs; sparse T cells; low antigen-presentation signals	Low immune-cell recovery may reflect true biology or technical dropout	Prioritize rapid tumor control with targeted therapy or intrathecal chemotherapy
T-cell-inflamed exhausted	Expanded TCR clones; CD8 effector genes; PD-1, TIM-3, LAG-3, TIGIT; moderate or falling ctDNA	T-cell abundance alone does not prove tumor specificity	Consider ICB, intrathecal nivolumab, cytokine-supported or cellular immunotherapy
Myeloid-suppressive	SPP1; MMP14; CSF1R; CCL2/CCR2; MIF/CD74; CD44/integrin programs	Myeloid states may vary by tumor type, treatment and sampling site	Combine tumor control with myeloid or chemokine-axis modulation
Cytokine-inflamed barrier-injury	IL-6; VEGF; complement activation; high protein; symptoms; steroid exposure	May reflect tumor progression, treatment injury or infection	Avoid blind immune escalation; consider anti-VEGF, cytokine-axis control and toxicity management
Molecularly resistant/therapy-adapted	Rising ctDNA despite treatment; resistance mutations; lineage plasticity; persistent CTCs	Plasma may miss CSF-private resistance; false negatives possible with low shedding	Switch or intensify targeted therapy; consider intrathecal or combination strategies
Low-cell/ctDNA-positive state	Low viable cells but detectable ctDNA or cfDNA alterations	Cell-based assays may fail because of low cellularity or processing delay	Use ctDNA as backbone; add targeted immune/protein assays when possible
CTC/CHC-enriched plastic state	High CTCs or circulating hybrid cells; partial EMT markers; adhesion/stemness programs	EpCAM-only capture may miss plastic tumor cells	Use label-flexible CTC platforms; consider therapies targeting adhesion, survival and resistance
Longitudinal response state	Falling ctDNA/CTCs; resolving cytokines; expanding productive TCR clones	Requires standardized serial sampling and matched blood controls	Continue effective therapy; use CSF trends to distinguish response, inflammation and relapse

CSF, cerebrospinal fluid; ctDNA, circulating tumor DNA; CTC, circulating tumor cell; TCR, T-cell receptor; PD-1, programmed cell death protein 1; TIM-3, T-cell immunoglobulin and mucin-domain containing-3; LAG-3, lymphocyte activation gene 3; TIGIT, T-cell immunoreceptor with immunoglobulin and ITIM domains; ICB, immune checkpoint blockade; SPP1, secreted phosphoprotein 1; MMP14, matrix metalloproteinase 14; CSF1R, colony-stimulating factor 1 receptor; CCL2, C-C motif chemokine ligand 2; CCR2, C-C chemokine receptor type 2; MIF, macrophage migration inhibitory factor; CD74, cluster of differentiation 74; CD44, cluster of differentiation 44; IL-6, interleukin-6; VEGF, vascular endothelial growth factor; EMT, epithelial-mesenchymal transition; EpCAM, epithelial cell adhesion molecule; CHC, circulating hybrid cell; cfDNA, cell-free DNA.

## 8 Therapeutic opportunities

### 8.1 Immune-state-based patient stratification

CSF profiling may create a future therapeutic opportunity through patient stratification, but this concept remains exploratory and should be built from measurable modules rather than abstract ecosystem labels. At present, LM management still relies largely on tumor type, driver mutation, performance status, MRI pattern, CSF cytology, and the systemic or intrathecal therapies available to the patient (2, 3, 121). These factors remain necessary, but they do not fully explain why patients with similar clinical profiles may respond in different ways. A practical CSF classification could begin with four readout groups: tumor burden and molecular evolution measured by ctDNA, CTCs or CHCs; malignant-cell and immune-cell states measured by scRNA-seq or proteomics; T cell function measured by cytotoxic transcripts, checkpoint expression and TCR clonality; and myeloid or barrier inflammation measured by SPP1/MMP14, CSF1/CSF1R, CCL2/CCR2, IL-6, VEGF, complement and related cytokines. For instance, a tumor-dominant ‘immune cold’ compartment may present elevated CSF ctDNA/CTC loads alongside scarce effector T cells and defective antigen presentation. A T cell-inflamed but exhausted state may combine clonally expanded TCRs, CD8 effector transcripts, PD-1/TIM-3/LAG-3 expression, and moderate or falling ctDNA. A myeloid-suppressive state may show SPP1, MMP14, CSF1/CSF1R, CCL2/CCR2, MIF/CD74, CD44 or integrin-associated programs. A cytokine-inflamed barrier-injury state may show high IL-6, VEGF, complement activation, symptoms, steroid exposure, and neurotoxicity risk. Each category should be viewed as a trial hypothesis until prospectively linked to response, neurological benefit, and toxicity.

Several additional states may be useful for future bedside classification. A low-cell, ctDNA-positive state cautions that cell-based assays may fail to detect active disease when CSF cellularity is low or processing is delayed. A CTC/CHC-enriched plastic state suggests partial epithelial-mesenchymal transition, adhesion, stemness, or hybrid tumor-immune characteristics that can be missed by EpCAM-only capture. A longitudinal response state should be defined by direction rather than a single value: falling ctDNA or CTCs, resolving cytokines, and expansion of productive TCR clones may support continued therapy, whereas discordant trends should trigger reassessment. These categories are deliberately tied to measurable CSF signals so that future studies can test, merge, or discard them (**Table 2**).

This proposal should not be understood as a validated clinical algorithm. It is better regarded as a trial-design framework. The suggested categories require prospective testing with predefined biomarkers, such as CSF ctDNA or MRD kinetics, CTC or circulating hybrid-cell burden, scRNA-seq-defined tumor and immune states, TCR clonality, cytokines, proteomics, and corresponding blood controls. Early cutoffs should be considered exploratory, as they will vary with assay, CSF volume, processing time, prior therapy, and disease distribution. The purpose of this framework is to help future studies determine which measurements predict treatment response and neurological benefit before they are reduced to a clinical decision tool (**Table 2**).

### 8.2 Rational combination strategies

Rational combinations should be based on the dominant measurable CSF axis rather than on the broad label of an ecosystem. Targeted therapy in oncogene-driven NSCLC LM may rapidly decrease tumor burden, but resistant clones and myeloid support may enable residual disease to persist (22, 78, 79). Intrathecal chemotherapy, anti-VEGF treatment or immunotherapy combinations might be acceptable in selected cases, but should not be used as a default option of treatment escalation (85, 93–95). For example, rising CSF ctDNA or CTCs with little immune activation would support testing tumor-directed intensification. Falling ctDNA with persistent cytokine elevation, neurological symptoms, or steroid requirement would instead suggest barrier injury, treatment-related inflammation, or cytokine-mediated toxicity and would require a different management approach.

Immunotherapy combinations also need pathway-level logic. ICB may be combined with radiotherapy to increase antigen release, with intrathecal delivery to improve CSF exposure, or with cellular therapy to place effector cells directly into the CSF (73, 76, 77, 96, 98). These approaches, however, are unlikely to work if antigen presentation is weak, effector T cells are absent, or macrophage-mediated suppression dominates the compartment. The most concrete candidate combination axes are SPP1-CD44/integrin, CSF1-CSF1R, CCL2-CCR2, MIF-CD74, VEGF, and IL-6/JAK/STAT3, because they connect tumor survival, barrier dysfunction, macrophage-like states, and T cell suppression (20, 22, 25, 44). Future trials should test whether a combination changes the biological axis it was designed to target: an ICB trial should document T cell activation or clonal expansion, a myeloid-targeting strategy should show macrophage reprogramming, and a targeted-therapy combination should reduce CSF tumor burden and delay resistant clones.

### 8.3 Clinical trial design for lung cancer LM

Clinical trials in lung cancer LM need designs that reflect the compartment where the disease occurs. For years, systemic therapy trials often excluded patients with LM because of poor prognosis, neurological instability, and the difficulty of measuring response (3, 24, 122). That exclusion slowed the field and left clinicians to rely on retrospective series, small cohorts, and case reports. Contemporary NSCLC LM cohorts and consensus reviews now support more systematic inclusion, especially for patients with stable performance status and measurable disease in CSF or on imaging (3, 46, 72).

The design of trials should involve paired blood and CSF samples, baseline and longitudinal MRI, neurological evaluation, and standardized response criteria (2–4, 122). Driver mutation, previous CNS therapy, MRI pattern, cytology or ctDNA status, and intended intrathecal or systemic route should be stratified. Linear and nodular LM are to be documented separately since they can vary in shedding of CSF, exposure to drugs, access to immune system, and measurability by imaging. Overall survival should not be the sole basis of trial endpoints. Relevant are progression-free survival, neurological progression, CSF molecular response, radiographic response, steroid requirement, treatment-related neurotoxicity, and quality of life. These endpoints will complicate trials but are required since LM impacts on both survival and neurological function.

### 8.4 Outcome assessment and neurofunctional endpoints

In patients with LM, the success of therapy cannot be based on tumor shrinkage. Outcome assessment includes neurological function, cognition, cranial nerve deficits, gait, headache, hydrocephalus, steroid exposure and quality of life (2, 3, 122). An intervention that extends survival but increases neurological toxicity may not be of much true value in a disease where symptoms frequently dictate day-to-day activity. In the same sense, a treatment that slows the progression of neurological deterioration and reduces the tumor burden in CSF might have clinical significance even in cases where radiographic response is small.

Future research should therefore incorporate patient-reported outcomes and neurofunctional scales. The CSF biomarkers can be used to interpret these endpoints, revealing whether the neurological deterioration is due to tumor progression, inflammation, hydrocephalus, treatment toxicity or a combination of processes simultaneously. This difference is particularly significant in immunotherapy and radiotherapy where inflammatory changes may resemble progression. CSF-guided therapy must manage compartmental disease with maintenance of neurological function.

### 8.5 Future perspective

The field appears to be moving from a singular focus on drug penetration toward a broader view of LM biology, but this shift remains incomplete. Getting into the CSF is important, but drug exposure alone may not explain all patterns of response or relapse. Lung cancer cells need to survive in nutrient-deprived environments, use barrier-associated niche structures, interact with suppressive macrophage-like programs, and keep evolving in response to treatment. Immune cells are present in some CSF samples, but available studies suggest that they may be converted into inflammatory, exhausted or myeloid-dominated states that fail to control tumor progression. These observations may help explain why single-modality approaches often have limited activity. They also highlight the importance of monitoring the CSF itself for understanding local disease progression and designing future treatment studies.

A future study approach could begin with simultaneous CSF and blood sampling at initial diagnosis, using ctDNA analysis together with immune-state assessment. The proposed decision framework would first define the dominant evidence-backed module rather than apply a generic ecosystem label: tumor-dominant molecular progression, T-cell-inflamed exhaustion, myeloid-suppressive remodeling, cytokine/barrier injury, low-cell ctDNA-positive disease, plastic CTC/CHC enrichment, or longitudinal response/resistance. Therapy could then be evaluated in relation to both the oncogenic driver and local CSF milieu, with repeated CSF sampling during early response and again at progression when clinically feasible. This approach may help identify patients more likely to benefit from targeted therapy, intrathecal chemotherapy, ICB, anti-VEGF combinations, cellular therapies, oncolytic or engineered local immunotherapies, or clinical trials. Equally important, it could help avoid unsupported treatment escalation by distinguishing whether progression is driven primarily by increasing tumor burden, immune suppression, cytokine-driven inflammation, or acquired molecular resistance. **Figure 3** should therefore be read as an exploratory decision framework that links specific CSF measurements to specific biological hypotheses, not as a validated treatment algorithm.

## 9 Conclusion

Lung cancer LM may be better understood as a compartmental tumor-immune process rather than only an anatomical sanctuary, but this model still requires clinical validation. The most useful synthesis is not the broad claim that CSF is an ecosystem, but the more testable claim that CSF disease can be decomposed into interacting tumor, myeloid, lymphoid, barrier-inflammatory, and molecular-monitoring modules. In the CSF niche, tumor cells are subjected to nutrient limitation, poor matrix attachment, barrier dependence and CNS border immunity. Malignant cells may in turn remodel this space through complement activation, metabolic adaptation, adhesion programs, lineage plasticity and recruitment of suppressive myeloid states. CSF immunity can also shift toward exhausted, regulatory, macrophage-rich or inflammatory but non-protective states, which may help explain why systemic biomarkers often fail to predict LM behavior. To advance the field, standardized CSF sampling and treatment studies incorporating ctDNA or MRD evaluation, CTC profiling, scRNA-seq, TCR tracking, cytokines, proteomics, and serial monitoring should test these modules prospectively. The primary challenge is to translate specific CSF measurements and pathway-level hypotheses into validated, clinically usable rules that can inform targeted therapy, intrathecal chemotherapy, immunotherapy, anti-VEGF therapy, radiotherapy, myeloid modulation, and engineered local therapy without compromising neurofunction and quality of life.

## Glossary

ADGRG1: Adhesion G protein-coupled receptor G1
ALK: Anaplastic lymphoma kinase
BCSFB: Blood-CSF barrier
BrM: Brain metastasis
CAR: Chimeric antigen receptor
C3: Complement component 3
CNS: Central nervous system
CSF: Cerebrospinal fluid
CTC: Circulating tumor cell
ctDNA: Circulating tumor DNA
CTLA-4: Cytotoxic T-lymphocyte-associated protein 4
DC: Dendritic cell
EGFR: Epidermal growth factor receptor
EGFR-TKI: Epidermal growth factor receptor tyrosine kinase inhibitor
ICB: Immune checkpoint blockade
ICI: Immune checkpoint inhibitor
IL: Interleukin
JAK: Janus kinase
LAG-3: Lymphocyte activation gene 3
LM: Leptomeningeal metastasis
MHC: Major histocompatibility complex
MRI: Magnetic resonance imaging
NK: Natural killer
NSCLC: Non-small cell lung cancer
PD-1: Programmed cell death protein 1
PD-L1: Programmed death-ligand 1
scRNA-seq: Single-cell RNA sequencing
STAT3: Signal transducer and activator of transcription 3
TCR: T cell receptor
TGF-β: Transforming growth factor-β
TIGIT: T cell immunoreceptor with immunoglobulin and ITIM domains
TIM-3: T cell immunoglobulin and mucin-domain containing-3
Treg: Regulatory T cell
VEGF: Vascular endothelial growth factor
PAV: Pre-analytical variation
MRD: Molecular residual disease
LOD: Limit of detection
IFN-gamma: Interferon-gamma
CHC: Circulating hybrid cell
B7-H3: B7 homolog 3
CCL2: C-C motif chemokine ligand 2
CCR2: C-C chemokine receptor type 2
CSF1: Colony-stimulating factor 1
CSF1R: Colony-stimulating factor 1 receptor
CXCL8: C-X-C motif chemokine ligand 8
CXCL9: C-X-C motif chemokine ligand 9
CXCL10: C-X-C motif chemokine ligand 10
CXCL12: C-X-C motif chemokine ligand 12
CXCL13: C-X-C motif chemokine ligand 13
CXCR2: C-X-C chemokine receptor type 2
CXCR3: C-X-C chemokine receptor type 3
CXCR4: C-X-C chemokine receptor type 4
EpCAM: Epithelial cell adhesion molecule
ITIM: Immunoreceptor tyrosine-based inhibitory motif
MIF: Macrophage migration inhibitory factor
MMP14: Matrix metalloproteinase 14
SPP1: Secreted phosphoprotein 1
TROP2: Trophoblast cell-surface antigen 2.
